# Derivation and validation of a blood biomarker score for 2-day mortality prediction from prehospital care: a multicenter, cohort, EMS-based study

**DOI:** 10.1007/s11739-023-03268-x

**Published:** 2023-04-20

**Authors:** Francisco Martín-Rodríguez, Fernando Vaquerizo-Villar, Raúl López-Izquierdo, Miguel A. Castro-Villamor, Ancor Sanz-García, Carlos del Pozo-Vegas, Roberto Hornero

**Affiliations:** 1 Faculty of Medicine, Universidad de Valladolid, Valladolid, Spain; 2 Advanced Life Support, Emergency Medical Services (SACYL), Valladolid, Spain; 3 Prehospital Early Warning Scoring-System Investigation Group, Valladolid, Spain; 4 Biomedical Engineering Group, Facultad de Medicina, Universidad de Valladolid, Av. Ramón y Cajal, 7, 47003 Valladolid, Spain; 5 CIBER-BBN, Centro de Investigación Biomédica en Red en Bioingeniería, Biomateriales y Nanomedicina, Valladolid, Spain; 6 Emergency Department, Hospital Universitario Rio Hortega, Valladolid, Spain; 7 Health Research Institute, Hospital de la Princesa, Madrid (IIS-IP), Spain; 8 Emergency Department, Hospital Clínico Universitario, Valladolid, Spain

**Keywords:** 2-day mortality, Blood biomarker score, Creatinine, Emergency medical services (EMS), Lactate, Partial pressure of carbon dioxide (pCO_2_)

## Abstract

Identifying potentially life-threatening diseases is a key challenge for emergency medical services. This study aims at examining the role of different prehospital biomarkers from point-of-care testing to derive and validate a score to detect 2-day in-hospital mortality. We conducted a prospective, observational, prehospital, ongoing, and derivation—validation study in three Spanish provinces, in adults evacuated by ambulance and admitted to the emergency department. A total of 23 ambulance-based biomarkers were collected from each patient. A biomarker score based on logistic regression was fitted to predict 2-day mortality from an optimum subset of variables from prehospital blood analysis, obtained through an automated feature selection stage. 2806 cases were analyzed, with a median age of 68 (interquartile range 51–81), 42.3% of women, and a 2-day mortality rate of 5.5% (154 non-survivors). The blood biomarker score was constituted by the partial pressure of carbon dioxide, lactate, and creatinine. The score fitted with logistic regression using these biomarkers reached a high performance to predict 2-day mortality, with an AUC of 0.933 (95% CI 0.841–0.973). The following risk levels for 2-day mortality were identified from the score: low risk (score < 1), where only 8.2% of non-survivors were assigned to; medium risk (1 ≤ score < 4); and high risk (score ≥ 4), where the 2-day mortality rate was 57.6%. The novel blood biomarker score provides an excellent association with 2-day in-hospital mortality, as well as real-time feedback on the metabolic-respiratory patient status. Thus, this score can help in the decision-making process at critical moments in life-threatening situations.

**Supplementary Information:**

The online version contains supplementary material available at 10.1007/s11739-023-03268-x.

## Introduction

Prehospital care has undergone a remarkable evolution in recent decades, providing advanced support at the scene, making decisions at an early stage, and optimizing comprehensive and appropriate care according to the individual case [1]. One of the key challenges for emergency medical services (EMS) must be to recognize life-threatening disease. To this end, the EMS-personnel can use a variety of different tools to guide decision-making, e.g., early warning scores, biomarkers, and predictive models [2, 3].

In this way, point-of-care testing (POCT) can provide a subtle awareness of pathophysiological changes that may otherwise go unnoticed, in addition, to sudden abnormalities that can activate a warning trigger and facilitate the decision-making process at the scene or en route [4, 5]. The implementation of POCT is an emerging trend in the clinical practice of prehospital care [6]. Technical advancements have resulted in rugged, portable, easy-to-use, and well-proven devices [7, 8], which can be used bedside by any EMS personnel, including paramedics [9].

Out-of-hospital, the first responders must perform lifesaving interventions, based on a limited data, and make a quick-decision at the site [6]. Typically, major diseases with worse prognosis will be easy to recognize, based on clinical presentation, abnormal vital signs, or the use of some easy-to-access diagnostic system in the prehospital care, e.g., electrocardiogram for diagnosis of acute coronary syndrome. However, other conditions may not show such clear warning signs of seriousness from the onset of the event and identifying potentially life-threatening diseases is at times a challenge for EMS [10]. Under these circumstances, POCT has demonstrated to be a useful support in the decision-making process, as it allows the EMS-personnel to know the status of the patient and hence apply an early treatment from the very beginning, without the need to wait for an in-hospital blood analysis to start with the urgent treatment [11].

By way of example, capillary-glucose testing revolutionized the out-of-hospital self-care of insulin-dependent diabetes patients. This procedure is widespread worldwide and provides accurate results for the bedside management of this pathology [12]. In this sense, the use of POCT in emergency departments (ED) and intensive care units (ICU) has gradually expanded as they can be time- and cost-saving interventions in certain applications, such as blood analysis [13, 14], whereas its implementation in the prehospital scope has started timidly. A range of POCT and a wide range of biomarkers can already be determined in a short-term timeframe bedside, e.g., venous blood gas, ions, creatinine, urea, glucose, lactate, coagulation, D-dimer, troponin, C-reactive protein, and procalcitonin [5, 13].

The primary endpoints of this study were to examine the role of different bedside biomarkers in adult patients, with the aim of deriving and validating a blood biomarker score to accurately detect all-cause in-hospital mortality within 2 days from the initial patient contact (2-day mortality), as well as to establish risk levels for 2-day mortality.

## Methods

### Study design and setting

This prospective, observational, prehospital, ongoing, and derivation—validation study was carried out in three Spanish provinces (Salamanca, Segovia, and Valladolid), with adults (> 18 years old) evacuated by ambulance and admitted to the ED between October 8, 2019, and October 27, 2021.

The EMS personnel involved in this study include six advanced life support (ALS) units, 38 basic life support (BLS) units, and four EDs (one minor general district hospital and three university tertiary hospitals). The BLS is composed of 2 emergency medical technicians (EMT) and the ALS is staffed by two EMTs, a physician, and an emergency registered nurse (ERN). Based on a first evaluation at the scene, the ALS physician decides for each patient if an evacuation is necessary and the appropriate type of ambulance (BLS or ALS) to be dispatched.

The institutional review committee of the Public Health Service validated the study protocol, which was prepared in adherence to the Helsinki Declaration. Two back-to-back studies were undertaken to accumulate data registered in the World Health Organization's International Clinical Trials Registry Platform (ISRCTN48326533 and ISRCTN49321933) according to STrengthening the Reporting of OBservational studies in Epidemiology.

### Population

During the study period, adult patients making a phone call to the emergency call center (1-1-2 calls) were examined for eligibility. Following the appropriate evaluation by the ALS physician, blood–venous analysis was performed in those patients referred to the ED (either ALS or BLS) and only these patients were included in the follow-up cohort. Each patient included in the study was evaluated at the scene by the ALS physician. Based on this objective and structured clinical evaluation and aided by complementary tests, a decision was made to decide the best treatment and/or destination for the patient (e.g., discharge on site or transfer to the hospital in BLS or in ALS).

Minors, terminally ill patients, pregnant, cardiorespiratory arrest, situations with potential risk for EMS-staff (e.g., weapons at the scene, aggressive subjects), and cases in which prehospital blood-analysis was not possible (e.g., device failure, inability to draw blood) were excluded. For all participants in the study, written informed consent was obtained.

### Outcome

The primary outcome was 2-day in-hospital mortality (includes all-cause mortality), in line with similar studies [15, 16]. All non-survivor caseloads were re-evaluated by the principal investigator.

### Data collection

All members of the research group received online theoretical training as well as on-site hands-on instruction on the system for data collection and the study endpoints.

Epidemiological information (age, sex, urban or rural area, ambulance type, and nursing home) was collected by an EMT. After the evaluation, when prehospital blood-analysis was indicated, the ERN performed an intravenous-line catheterization and blood sampling. A total of 23 ambulance-based biomarkers were directly obtained or indirectly calculated from blood analysis: pH, partial pressure of carbon dioxide (pCO_2_), partial pressure of oxygen (pO_2_), bicarbonate, blood base excess, oxygen saturation (cSO_2_), sodium, potassium, calcium, chlorine, total carbon dioxide content (TCO_2_), hematocrit, hemoglobin, extracellular base excess, glucose, lactate, creatinine, GAP anion, urinary anion, K anion, osmolarity, urea, and blood urea nitrogen (BUN). The ambulance physician registered the prehospital diagnosis group based on the International Classification of Diseases 11th Revision.

In all the patients, the blood analysis was carried out using the epoc^®^ POCT Blood Analysis System. Each analysis was performed with a self-calibrating card with control of expiration dates, serial numbers, and batch numbers. After the analysis card has been inserted into the device and self-calibration has been performed, the venous sample is deposited and, in 45 s, the results are displayed on the screen of epoc^®^ system. epoc® has been implemented and validated in an extensive variety of clinical conditions to check the bedside parameters and their subsequent confirmation by conventional analytical equipment [8, 13, 17, 18].

Finally, after the end of the follow-up period go of 2 days (since the prehospital index event) and by reviewing the hospital electronic medical record, a co-researcher collected clinical records (hospital inpatient, in-hospital 2-day mortality, and ICU-admissions).

The outcomes were blinded to the clinicians involved in the data gathering. The analytical data was transferred via Wi-Fi from the epoc^®^ Blood Analysis System to the principal investigator's computer. The compiled information was recorded in a database generated with the IBM SPSS Statistics for Apple version 20.0 software.

### Score calculation

Before the development and validation score, the cohort was randomly assigned into a derivation cohort (60%) and a validation cohort (40%). The blood biomarker score was fitted using the derivation group and its accuracy is evaluated using the validation group. First, a feature selection stage was developed to obtain the variables that constitute the blood biomarker score. In this respect, the fast correlation-based filter (FCBF), an automated feature selection algorithm [19], was used to select an optimum subset of ambulance-based biomarkers related to 2-day mortality. The biomarker score was built using this optimum blood biomarker subset in the derivation cohort as follows [20]:

(i) *Variable transformation.* The biomarkers from the optimum subset are transformed into categorical variables. Intervals from each category were then obtained using locally estimated scatterplot smoothing (LOESS) curves (see Supplementary Material).

(ii) *Weights calculation.* A logistic regression (LR) model is fitted using these categorical variables to predict 2-day mortality (survivors vs. non-survivors). Score weights for each interval of the categorical biomarkers were then obtained as the beta coefficients of the LR model.

(iii) *Score calculation.* The final score is obtained from the sum of the weights in each categorical variable.

### Statistical analysis

A full variable-by-variable analysis was then conducted for blood biomarkers, demographics, and clinical baseline patients’ characteristics using logic, range, consistency, and missing data tests, resulting in a total of 32 variables. Absolute values and percentages were employed for categorical variables and median and interquartile ranges (IQR) for continuous variables (the Shapiro–Wilk test showed a non-normal distribution). The Chi-square and Mann–Whitney *U* tests were used for the comparison of qualitative and quantitative variables with 2-day mortality (survivors *vs*. non-survivors), respectively.

The overall predictive validity of the blood biomarker score was assessed by means of the area under the curve (AUC) of the receiver operating characteristic (ROC) in the validation cohort, as well as the Brier score and R^2^. In addition, the diagnostic ability of the score at each cutoff point was evaluated by means of sensitivity (Se), specificity (Sp), positive predictive value (PPV), negative predictive value (NPV), positive likelihood ratio (LR +), negative likelihood ratio (LR-), and odds ratio (OR). These statistics were used to establish risk levels for 2-day mortality. 95% confidence intervals (95% CI) were obtained for each of these statistics using Wilson score [21]. A decision curve analysis was also performed to show the clinical applicability of the blood biomarker score [22].

The data were analyzed using our own codes and base functions in R 4.1.2 (the R Foundation for Statistical Computing, Vienna, Austria), Matlab R2018a (The MathWorks Inc., Natick, MA, USA), and Python 3.6.9 (the Python Software Foundation, Wilmington, DE, USA).

## Results

### Patient's baseline

Overall, 3925 adult patients with acute disease were screened by EMS and subsequently referred to the ED, resulting in an analysis cohort of 2806 cases according to the exclusion criteria (see Supplemental Fig. 1). Non-survivors represented 5.5% (154 cases), with a raised median age than survivors [median and IQR: 77 (62–85) *vs*. 67 (50–80) years] with the same sex distribution, predominantly from urban areas, and approximately 1-in-3 patients arriving via a nursing home. Cardiovascular (53 cases, 34.4%), trauma and injury (31 cases, 20.1%), neurology disease (20 cases, 13.0%), and infection (20 cases, 13.0%) are the main causes of the reported 2-day mortality (deliberate self-harm is classified under poisoning or injury as appropriate), with an ICU-admission rate (including all-cause mortality) of 42.2% (see Table 1). The derivation cohort was composed of 1684 cases (60%, 1591 survivors and 93 non-survivors), whereas the validation cohort had 1122 cases (40%, 1061 survivors and 61 non-survivors). The ratios of survivors/non-survivors (2-day mortality) in both cohorts remained similar: 1591/63 (17.11) in the derivation cohort and 1061/61 (17.39) in the validation cohort.

**Fig. 1:**
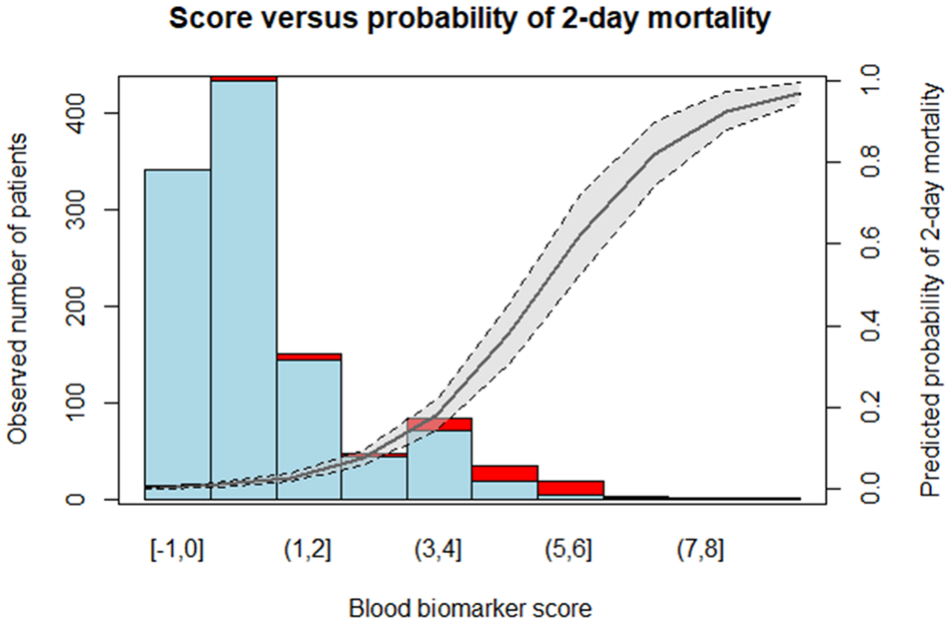
Score vs. real and predicted probability of 2-day mortality. The grey area of the trend line corresponds to 95% confidence interval of the predicted probability of death (trend line). The bars correspond to the number of patients of the validation cohort alive (blue) or death (red)

**Table 1:** Clinical and biomarkers baseline patients’ characteristics

	Total	Survivors	Non-survivors	*p-*value^b^
No. (%) with data^a^	2806	2652 (94.5)	154 (5.5)	NA
Age, year	68 (51–81)	67 (50–80)	77 (62–85)	< 0.001
Sex, female	1186 (42.3)	1121 (42.3)	65 (42.2)	0.988
Advanced life support	1854 (66.1)	1720 (64.9)	134 (87)	< 0.001
Zone, urban	2040 (72.7)	1936 (73)	104 (67.5)	0.139
Nursing homes	332 (11.8)	286 (10.8)	46 (29.9)	< 0.001
Prehospital blood analysis				
pH	7.38 (7.33–7.42)	7.39 (7.34–7.43)	7.23 (7.01–7.36)	< 0.001
pCO_2_, mmHg	40.2 (34–47.4)	40.1 (34.1–45.8)	49.8 (40.3–71.3)	< 0.001
pO_2_, mmHg	35.2 (25.1–45.3)	36.7 (26.3–45.7)	24.8 (18.5–36.4)	< 0.001
Bicarbonate, mEq	24.1 (22.1–26.7)	24.3 (22.5–26.7)	20.5 (15.5–24.2)	< 0.001
BE (efc), mmol/L	0.6 (-2.8, 2.1)	0.8 (-2.1, 2.1)	-4.5 (-12.5, -0.3)	< 0.001
cSO_2_, %	56.7 (40.5–70.8)	57.1 (43.2–71.3)	39.1 (23.7–59.0)	< 0.001
Sodium, mmol/L	139 (137–140)	139 (137–140)	138 (133–143)	0.619
Potassium, mmol/L	4.1 (3.8–4.5)	4.1 (3.8–4.5)	4.1 (3.7–4.9)	0.181
Calcium, mmol/L	1.14 (1.07–1.19)	1.14 (1.08–1.19)	1.04 (0.95–1.19)	< 0.001
Chlorine, mmol/L	103 (100–106)	103 (100–105)	103 (100–107)	0.509
TCO_2_, mmol/L	25.2 (22.6–28.3)	25.2 (22.8–28.1)	24.0 (20.5–28.7)	0.110
Hematocrit, %	41 (38–45)	42 (38–45)	39 (32–44)	< 0.001
Hemoglobin, g/dL	14.1 (12.6–15.6)	14.2 (12.7–15.7)	12.9 (11.1–15.1)	< 0.001
BE (b), mmol/L	0.7 (-2.9, 2.1)	0.8 (-2.4, 2.1)	-4.8 (-12.4, -0.9)	< 0.001
Glucose, mg/dL	130 (106–165)	127 (104–161)	176 (138–241)	< 0.001
Lactate, mmol/L	2.08 (1.24–3.21)	1.96 (1.18–3.07)	6.92 (4.65–9.62)	< 0.001
Creatinine, mgr/dL	0.94 (0.76–1.23)	0.91 (0.76–1.17)	1.97 (1.09–2.79)	< 0.001
GAP anion, mmol/L	11.2 (8.1–14.4)	11.2 (8.1–14.4)	13.7 (8.8–19.3)	< 0.001
Urinary anion, mmol/L	40 (37–42.1)	40.0 (37.1–42.7)	39.1 (34.9–43.6)	0.323
Potassium anion, mmol/L	15.3 (12.3–18.7)	15.3 (12.3–18.6)	18.4 (13.2–23.8)	< 0.001
Osmolarity, mOsm/Kg	291 (286–297)	292 (287–297)	298 (291–307)	< 0.001
Urea, mg/dL	36.6 (27.6–50.6)	36.1 (27.0–49.8)	62.5 (38.9–85.4)	< 0.001
BUN, mg/dL	17.1 (12.8–23.6)	16.8 (12.6–23.3)	29.1 (18.1–39.9)	< 0.001
Prehospital diagnosis				
Cardiovascular	1017 (36.2)	964 (33.3)	53 (34.4)	
Neurology	490 (17.5)	470 (17.7)	20 (13.1)	
Trauma and injury	470 (16.7)	439 (16.6)	31 (20.1)	
Respiratory	199 (7.1)	183 (6.9)	16 (10.4)	
COVID-19	104 (3.7)	93 (3.5)	11 (7.1)	
Poisoning	247 (8.8)	242 (9.1)	5 (3.2)	
Infection	185 (6.6)	165 (6.2)	20 (13)	
Digestive	125 (4.5)	120 (4.5)	5 (3.2)	
Others^c^	73 (2.6)	69 (2.6)	4 (2.6)	0.349
Hospital outcomes				
Hospital-inpatient	1626 (57.9)	1478 (55.7)	145 (100)	< 0.001
ICU-admission	317 (11.3)	252 (9.5)	65 (42.2)	< 0.001

*pCO*_*2*_: partial pressure of carbon dioxide, *pO*_*2*_: partial pressure of oxygen, *BE:* base excess, *cSO*_*2*_: oxygen saturation, *TCO*_*2*_: total carbon dioxide content, *BUN:* blood urea nitrogen, *ICU:* intensive care unit, *NA:* NOT applicable

^a^Values expressed as total number (fraction) and medians [25 percentile-75 percentile], as appropriate

^b^The Mann–Whitney *U* test or Chi-squared test was used as appropriate

^c^Other pathology: endocrine, genitourinary, diseases of the blood and the immune system

### Score optimization

An optimum subset composed of pCO_2_, lactate, and creatinine was selected in the feature selection stage using the derivation cohort. These 3 biomarkers provide relevant and non-redundant information to detect 2-day in-hospital mortality. For each biomarker, the intervals associated with risk categories were obtained through a visual analysis of the slope of the LOESS curves (see Supplemental Fig. 2) and the blood biomarker score was subsequently fitted using these categorical variables.

**Fig. 2:**
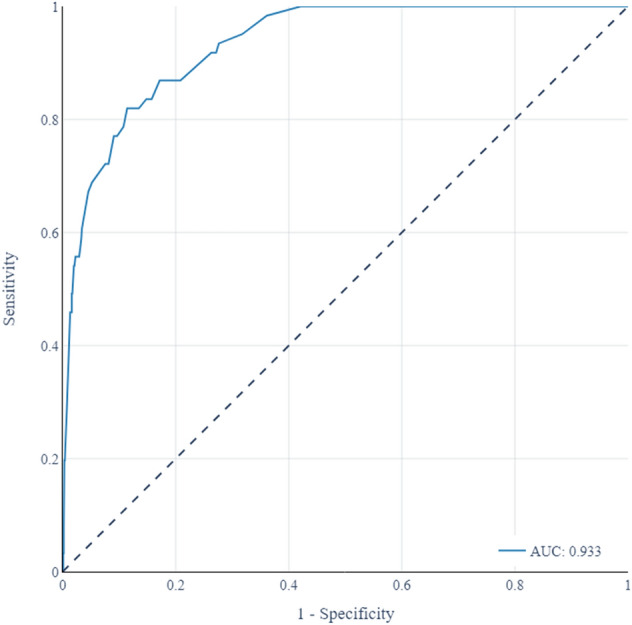
Receiver operational curve (ROC) for the blood biomarker score

## Score accuracy

Table 2 shows the scoring system developed, with the corresponding ratings for each ambulance-based biomarker, whereas Supplemental Table 1 shows the odd ratios of the score for each interval of these biomarkers. Notice that higher weights and odd ratios of the blood biomarker score were obtained with increasing values of pCO_2_, lactate, and creatinine. The distribution according to the score and the probability of projected undergoing 2-day mortality are displayed in the histogram included Fig. 1, whereas Supplemental Fig. 4 shows violin plots of the score in survivors and non-survivors groups in the validation cohort. Only 8.2% of non-survivors have a score lower than 1 point, showing a 2-day mortality rate of 57.6% for a score over 4 points.

**Table 2:** Blood biomarker score for 2-day mortality prediction from ambulance-based variables

Variable	Interval	Score
pCO_2_, mmHg	0–25	0
	25–40	− 0.67
	41–55	0.12
	56–125	0.47
	> 125	3.84
Lactate, mmol/L	0–3	0
	3.1–4.7	1.22
	4.8–9	3.12
	9.1–13	3.25
	13.1–16	3.48
	> 16	3.79
Creatinine, mgr/dL	0–1.5	0
	1.6–3	1.34
	3.1–4.5	1.48
	4.5–6.5	1.59
	6.6–9	3.53
	> 9	3.87

*pCO*_*2*_: partial pressure of carbon dioxide

*Interpretation*: (−1, 1) low risk; (1,4); intermediate risk; ≥ 4 high risk

The predictive validity of the score was calculated by estimating AUC (see Fig. 2), Brier score, and R^2^ in the validation cohort, returning an AUC of 0.933 (95% CI 0.841–0.973, *p* < 0.001), a Brier score of 0.034 (95% CI 0.010–0.114), and a R^2^ of 0.455 (95% CI 0.336–0.579). The calibration curve of the blood biomarker score in the validation cohort can be seen in the Supplemental Fig. 3, together with additional predictive statistics. For the sake of completeness of the analysis, supplementary material also includes a comparison of the performance of different blood biomarker design strategies (see Supplemental Table 2), a comparison of the blood biomarker score with a biomarker score that also integrates age and sex (Supplemental Table 3), and a comparison of the blood biomarker score with a score based on decision trees (Supplemental Table 4).

**Fig. 3:**
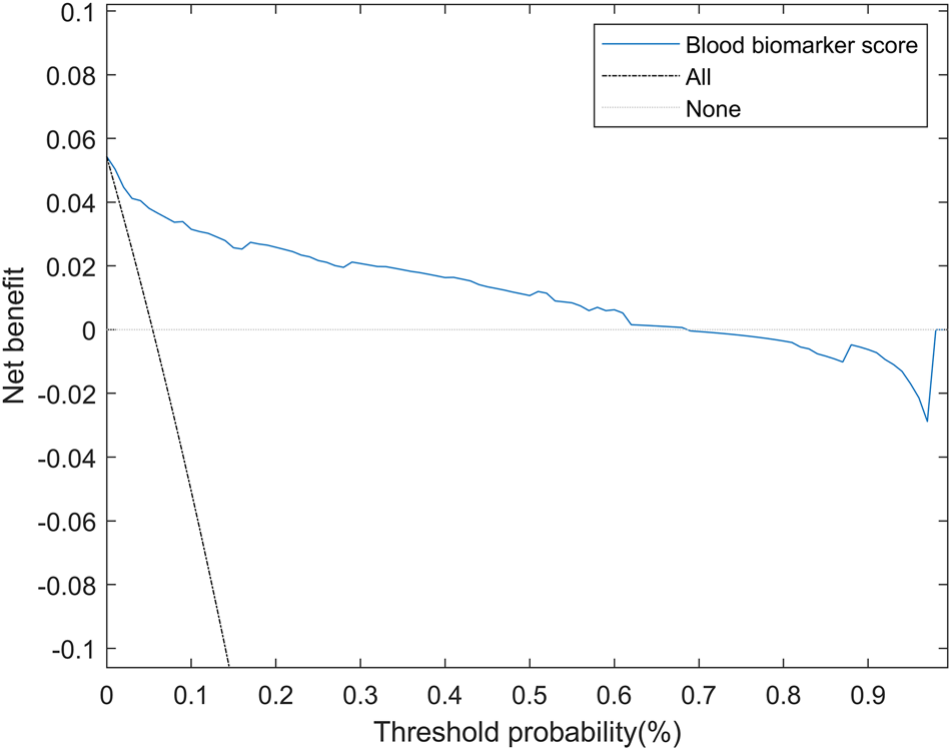
Decision curve of the blood biomarker score

Table 3 summarizes the model statistics for each integer cutoff of the blood biomarker score. A score lower than 1 point has an excellent sensitivity of 91.8 (95% CI 82.2–96.5) and a specificity of 73.0 (95% CI 60.8–82.6), together with a negative likelihood ratio of 0.11 (95% CI 0.06–0.22). Conversely, a result over or equal to 4 points has a sensitivity of 55.7 (95% CI 43.3–67.5) and an excellent specificity of 97.6 (95% CI 90.2–99.5), in aggregate with an excellent positive likelihood ratio of 23.7 (95% CI 8.0–74.6). Hence, the following risk levels can be established: low risk (− 1, 1), intermediate risk (1,4), and high risk (≥ 4).

**Table 3:** Statistical details of the blood biomarker score according to each cutoff

Score cutoff	Se (%)^a^	Sp (%)^a^	PPV (%)^a^	NPV (%)^a^	LR+ ^a^	LR−^a^	OR^a^
− 1	100 (94.1–100)	0 (0–5.9)	5.4 (2.0–14.2)	0 (0–5.9)	1 (1–1.1)	0 (0–0.06)	N.D
0	100 (94.1–100)	29.6 (19.6–42.0)	7.5 (3.2–19.7)	100 (94.1–100)	1.4 (1.2–1.7)	0 (0–0.06)	N.D
1	91.8 (82.2–96.5)	73.0 (60.8–82.6)	16.4 (9.1–27.6)	99.4 (92.9–99.9)	3.4 (2.4–5.1)	0.11 (0.06–0.22)	30.4 (8.9–110)
2	82.0 (70.5–89.6)	86.7 (76.0–93.1)	26.2 (16.8–38.4)	98.8 (92.0–99.8)	6.2 (3.6–11.0)	0.21 (0.12–0.33)	29.7 (8.8–106)
3	77.0 (65.1–85.8)	90.9 (81.1–95.9)	32.9 (22.4–45.4)	98.6 (91.6–99.8)	8.5 (4.5–17.1)	0.25 (0.16–0.37)	33.7 (9.3–130)
4	55.7 (43.3–67.5)	97.6 (90.2–99.5)	57.6 (45.1–69.2)	97.5 (89.9–99.4)	23.7 (8.0–74.6)	0.45 (0.33–0.58)	52.2 (10.9–265)
5	29.5 (19.6–41.9)	99.3 (92.9–99.9)	72.0 (59.7–81.7)	96.1 (87.9–98.8)	44.7 (10.4–205)	0.71 (0.59–0.81)	63.0 (11.6–365)
6	6.6 (2.6–15.7)	99.8 (93.7–100)	66.7 (54.2–77.2)	94.9 (86.3–98.2)	34.8 (9.4–136)	0.94 (0.85–0.98)	37.2 (9.7–152)
7	3.3 (0.9–11.1)	99.9 (93.9–100)	66.7 (54.2–77.2)	94.7 (86.0–98.1)	34.8 (9.4–136)	0.97 (0.89–0.99)	35.9 (9.6–144)
8	0 (0–5.9)	99.9 (93.9–100)	0 (0–5.9)	94.6 (85.8–98.0)	N.D	1 (0.94–1)	N.D

*Se* sensitivity, *Sp* specificity, *PPV* positive predictive value, *NPV* negative predictive value, *LR+* positive likelihood ratio, *LR–* negative likelihood ratio, *OR* odds ratio, *N.D* not defined

^a ^Bracketed number indicate 95% confidence interval

Figure 3 shows the decision curve of the blood biomarker score in the validation cohort. It can be observed that the net benefit of the proposed score is higher than the strategy of labelling all subjects at risk for 2-day mortality (“All” approach: black line) for all the probability thresholds, as well as greater than the strategy of not labelling any patient at risk for 2-day mortality (“None” approach: grey line) for threshold probabilities between 0 and 68% (score values lower than 5.6).

## Discussion

In this study, we derived and validated a blood biomarker score, available and based on ambulance-based prehospital biomarkers. Our results suggest that the proposed bedside score, involving pCO_2_, lactate and creatinine, provides an excellent association with 2-day in-hospital mortality. Biomarkers are definitively common in clinical practice, and the application of scores is a convenient way to accurately manage the interpretation of these indicators [11]. However, to our knowledge, this is the first prehospital score that integrates pCO_2_, lactate, and creatinine upfront in acute disease (on scene or *en route*) to screen patients with a high risk of impairment and a potential associated risk for 2-day mortality.

Several studies have shown a remarkable performance of prehospital lactate as a predictor of early mortality. Swan et al. [23] reported an AUC for unplanned-ICU admission and mortality of 0.676 and 0.633, respectively, albeit with a relatively limited cohort. Martín et al. [3] reported an AUC of 0.867 for 2-day mortality. Figueira et al. [24] developed a score for use in patients listed for liver transplantation (including lactate and creatinine), with an AUC of 0.835 to detect in-hospital mortality. By the same way, pCO_2_ has been used in several scores. Meng et al. [25] analyzed the venoarterial pCO_2_ as a predictor of organ injury in patients with severe acute pancreatitis and Hedstrom et al. [26] evaluated the aggregate value of pCO_2_ in Silverman Andersen respiratory severity score to anticipate the potential requirements to respiratory support in newborns. Our score outperforms these outcomes, with an AUC of 0.933, a Brier score of 0.034, and a R^2^ of 0.455, exhibiting an excellent prognostic ability for 2-day mortality.

Regarding clinical practice, the use of scoring systems is a proven strategy in multiple clinical settings for a broad spectrum of diseases and has already implemented in prehospital care to facilitate the decision-making process on a regular ongoing basis [16]. In this respect, the decision curve analysis of our score, with a net benefit greater than “All” and “None” strategies, showed its clinical applicability. Lactate is one of the three biomarkers that make up the score and plays a well-studied role in risk-stratification in prehospital care [3, 23]. Similarly, elevated lactate levels correlate with increased morbidity–mortality and a significant excess of in-patient hospital and non-unplanned ICU-admissions [27]. Tissue hypoperfusion implies insufficient energy production and, as a concomitant consequence, an accumulation of metabolic by-products, including lactate. Under specific physio-pathological circumstances, e.g., sepsis, trauma, poisonings, burns, and acute cardio-vascular diseases, hyperlactatemia (> 4 mmol/L) is linked to impaired outcomes. In contrast, lactate clearance is a robust reporter of metabolic up-regulation [28, 29]. Creatinine is the second endpoint included in the score. The measurement of this biomarker is widespread worldwide in all routine blood tests and represents an alarming prompt indicator of kidney function and an excellent indicator of the build-up of metabolic by-product waste products, with related complications and possible dysfunction of additional organs or systems [30]. Finally, the model incorporated pCO_2_, a gaseous portion with a considerable diffusion capacity, showing the effectiveness of alveolar ventilation. pCO_2_ changes (hypercapnia or hypocapnia) have a key regulatory role in the acid–base balance, representing one of the buffer systems (in addition to bicarbonate) for the acidosis regulation, via the elimination of carbon dioxide by breathing [31].

A crucial point for prehospital care is the rapid time to pinpoint life-threatening disease. In this sense, the benefits provided by POCT are particularly noteworthy. As a result of ongoing technological innovations, EMS can now have portable, robust, built-in data transmission capabilities and trustworthy devices, which can be used bedside with confidence for rapid blood-test [32]. During critical situations, as in life-threatening diseases, EMS-providers must make quick-decisions. These decisions must be based on clinical and scientific evidence, guided by objective and systematized clinical evaluation, and supported by complementary diagnostic tests, which can be carried out on the site [33]. To this end, biomarkers in general and the suggested score could target and guide the timely action of EMS, particularly in patients without serious abnormalities in standard vital signs. Nevertheless, when performing a routine blood-test, we can identify fine changes, which when analyzed together, and with the appropriate weightings according to a score, can show us the current-condition and the real risk of deterioration of the patient.

### Limitations

Our study is not free on limitations. Regarding the data collection, it is important to first note that it is a pure convenience sample. To control for possible bias, data gathering was performed non-stop, 24/7, for two uninterrupted years, in urban, suburban, and rural areas, and in several separate sites and station ambulances, to make the overall sample as broadly representative. Future studies should assess the blood biomarker score in a large database with a more balanced proportion between survivors and non-survivors, as well as assessing the effect of incorporating the distance from specialty care and the nursing home status to the blood biomarker score. In addition, a singular POCT was selected, recognizing that there are alternative commercially devices with different sets of testing capabilities. The epoc^®^ was selected for several reasons: implementation in the regional EMS, familiarity with its use by the personnel, automatic self-calibration of individual analysis cards, portability, storage, and, finally, because the device provided the most of biomarkers with a single reagent of all POCT evaluated. Third, the principal dependent outcome was 2-day in-hospital mortality (includes all-cause mortality), a finding uninterpretable. All cases of non-survivor were double-checked by the entering researcher and secondarily verified by the principal investigator. In this respect, another future goal would be further validation of our proposed methodology to estimate survival at more times, including 7-day, 14-day, and 30-day mortality from the initial patient contact.

Regarding the study type and setting, we must first point out that this is a non-randomized, non-controlled study. Blood analysis at the scene was left to the discretion of the ALS physician, who based on the objective and structured clinical assessment, routine complementary tests (e.g., vital signs, electrocardiogram) decides whether to perform the prehospital analysis. This bias should be described, although we believe that the effect has been negligible, since of all the patients evacuated to the ED, 92.4% underwent analysis, excluding cases of patients with psychiatric pathology, objectively mild cases, or social problems. Another limitation is that POCT are usually employed in ALS but their global deployment in emergency medical services (EMS) has not been yet carried out. Notwithstanding, due to its robustness, usability, autocalibration, and ease of transportation, POCT can be used bedside by any EMS personnel, including paramedics. In this respect, future studies should compare the performance of the blood biomarker score in units with different technical capacity, as well as evaluate if there is any difference in the performance of the score between BLS and ALS. Finally, part of the study was carried out during the ongoing COVID-19 pandemic, an outbreak that requires further epidemiological analysis to determine the true impact of the pandemic on mortality rates and associated influence on cases of acute diseases, as well as its influence on the biomarkers constituting the score.

## Conclusions

The novel blood biomarker score, composed of three correlated prehospital variables (creatinine, lactate and pCO2), provides an excellent association with 2-day in-hospital mortality. The proposed score delivers real-time feedback on the metabolic–respiratory status of the patient and can help in the decision-making process at critical moments in life-threatening situations.

EMS systems should focus on incorporating POCT, portable diagnostic solutions with an unmatched understanding of patients at high risk of clinical impairment, as part of everyday workflows.

## Supplementary Information

Below is the link to the electronic supplementary material.

Supplementary file1 (CSV 369 KB)

Supplementary file2 (PDF 860 KB)

## Data Availability

Supplementary file 1 (.CSV format) contains the date, amublance-based biomarkers, outcome (2-day mortality), and cohort group (derivation/validation) of each patient of the database employed in this study.
