# Supplementary material for: Derivation and validation of a blood biomarker score for 2-day mortality prediction from prehospital care: a multicenter, cohort, EMS-based study
Source: Intern Emerg Med. 2023 Apr 20;18(6):1797–806. doi: 10.1007/s11739-023-03268-x (PMC10116443; doi:10.1007/s11739-023-03268-x)
Supplement: Supplementary file 2 — Supplementary file2 (PDF 860 KB) [file 11739_2023_3268_MOESM2_ESM.pdf]

Derivation and validation of a blood biomarker score for 2-day mortality prediction from prehospital care: a multicenter, cohort, EMS-based study

Francisco Martín-Rodríguez, PhD[a,b,c]; Fernando Vaquerizo-Villar, PhD[d,e]; Raúl López-Izquierdo, PhD[a,,c,f]; Miguel A. Castro-Villamor, PhD[a,c]; Ancor Sanz-García, PhD[c,g]; Carlos del Pozo Vegas, PhD[a,c,h]; Roberto Hornero, PhD[d,e]

[a] Faculty of Medicine, Universidad de Valladolid, Valladolid, Spain.

[b] Advanced Life Support, Emergency Medical Services (SACYL), Valladolid, Spain.

[c] Prehospital early warning scoring-system investigation group, Valladolid, Spain.

[d] Biomedical Engineering Group, Universidad de Valladolid, Valladolid, Spain.

[e] CIBER-BBN, Centro de Investigación Biomédica en Red en Bioingeniería, Biomateriales y Nanomedicina, Valladolid, Spain.

[f] Emergency Department, Hospital Universitario Rio Hortega. Valladolid, Spain.

[g] Data Analysis Unit, Health Research Institute, Hospital de la Princesa, Madrid (IIS-IP), Spain.

[h] Emergency Department, Hospital Clínico Universitario, Valladolid, Spain.

Corresponding author:

Dr. Fernando Vaquerizo-Villar

Address: Facultad de Medicina, Av. Ramón y Cajal, 7, 47003, Valladolid, Spain.

e-mail: fernando.vaquerizo@gib.tel.uva.es

Phone: (+34) 983185570

Supplemental Information

1. Study population

Supplemental Fig. 1 shows the flowchart of the study population.

Supplemental Fig. 1. Flowchart of the study population.

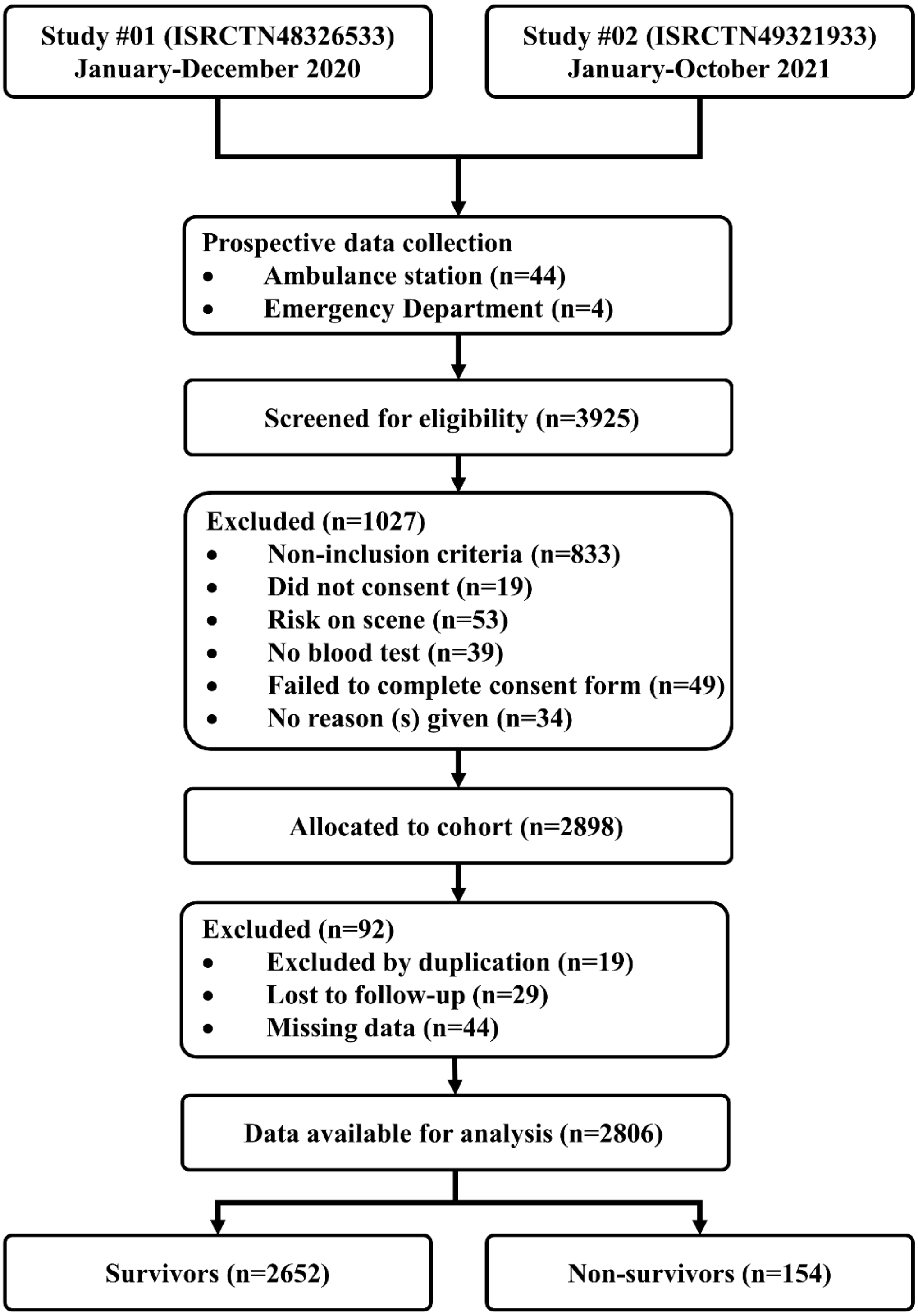

2. Locally estimated scatterplot smoothing (LOESS) curves

Following the recommendations of Zhang et al (2017), locally estimated scatterplot smoothing (LOESS) curves were used to convert the selected blood biomarkers, which are continuous variables, into categorical variables. LOESS is a smoothing technique that plots the probability of the outcome versus the numeric variable of interest. Then, cutoff points of the numeric variable are identified as those points where there is a change in the slope of the curve. These cutoff points are used to divide the numeric variable into risk categories (intervals). In this study, LOESS curves have been used to display the probability of 2-day mortality versus the value of partial pressure of carbon dioxide (pCO_2), lactate, and creatinine, as shown in Supplemental Fig. 2. By examining the slope of these curves, the selected biomarkers have been divided into the following categories:

- pCO_2: 0-25, 25-40, 40-55, 55-125, 125-∞.
- Lactate: 0-3, 3-4.7, 4.7-9, 9-13, 13-16, 16-∞.
- Creatinine: 0-1.5, 1.5-3, 3-4.5, 4.5-6.5, 6.5-9, 9-∞

3. Blood biomarker score calculation and interpretation

A biomarker score based on logistic regression (LR) was designed and validated to predict 2-day mortality from an pCO_2, lactate, and creatinine. A LR model is fitted using categorical biomarkers to predict 2-day mortality. For each category of the input biomarkers, score weights are obtained as the beta coefficients from the model. The final blood biomarker score is then obtained as the sum of the weights of the corresponding category of each biomarker.

Supplemental Fig. 2. LOESS curve plotting the probability of 2-day mortality against A) pCO_2, B) lactate, C) creatinine. The X-axis represents the values of the continuous variable, and the Y-axis represents the probability of outcome from 0 to 1. The blue line represents the LOESS smoothing curve for the probability of outcome and the gray area is the 95% confidence interval. Jittering has been used to add a small amount of random variation to the location of each point in order to handle overplotting.

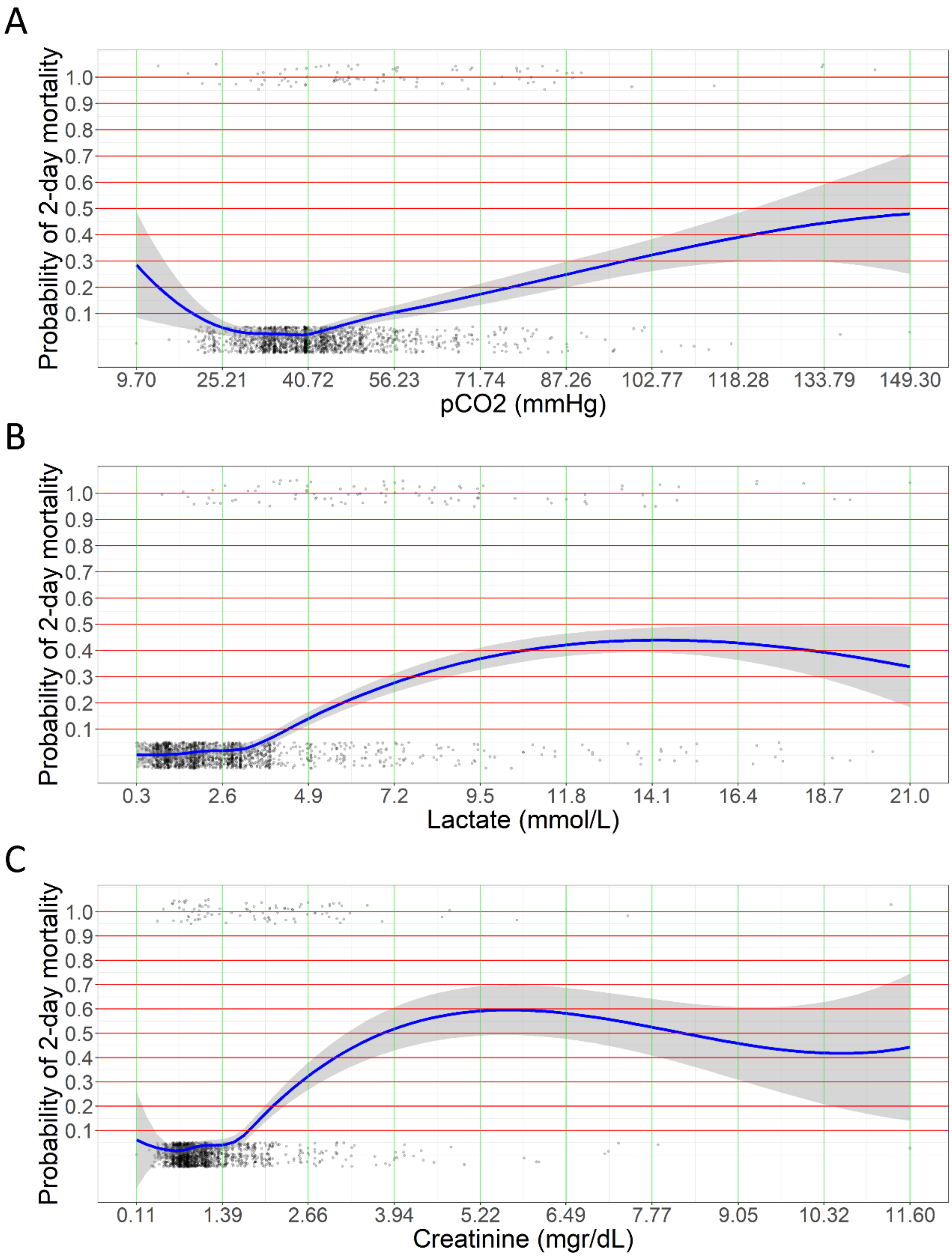

Supplemental Table 1 shows the odds ratio of the LR model for each category of the three biomarkers. It can be seen that 2-day mortality risk is substantially increased for values of pCO_2 higher than 125 mmHg. In the case of lactate, values higher than 3 imply an increase 2-day mortality risk, especially when lactate>4.7 mmol/L. Regarding creatinine, values higher than 1.5 increase the likelihood of 2-day mortality probability, being this likelihood very high when creatinine>9 mgr/dL. Supplemental Fig. 3 shows the calibration curves of the predicted probability against the actual probability in the validation cohort. Similarly, the distribution of the blood biomarker score among survivors and non-survivors can be observed in the violin plots displayed in Supplemental Fig. 4. Notice that most of survivors have a score lower than 1, whereas most of non-survivors have a score higher than 3.

Supplemental Table 1. Odd ratios of the blood biomarker score

Variable	Interval	Odds ratio
pCO2, mmHg	0-25	1
	25-40	0.51
	41-55	1.13
	56-125	1.60
	>125	46.53
Lactate, mmol/L	0-3	1
	3.1-4.7	3.39
	4.8-9	22.64
	9.1-13	25.79
	13.1-16	32.46
	>16	44.26
Creatinine, mgr/dL	0-1.5	1
	1.6-3	3.82
	3.1-4.5	4.39
	4.5-6.5	4.90
	6.6-9	34.12
	>9	47.94

Abbreviations: pCO2: partial pressure of carbon dioxide.

Supplemental Fig. 3 Calibration curve of the blood biomarker score. A variety of statistics are also included.

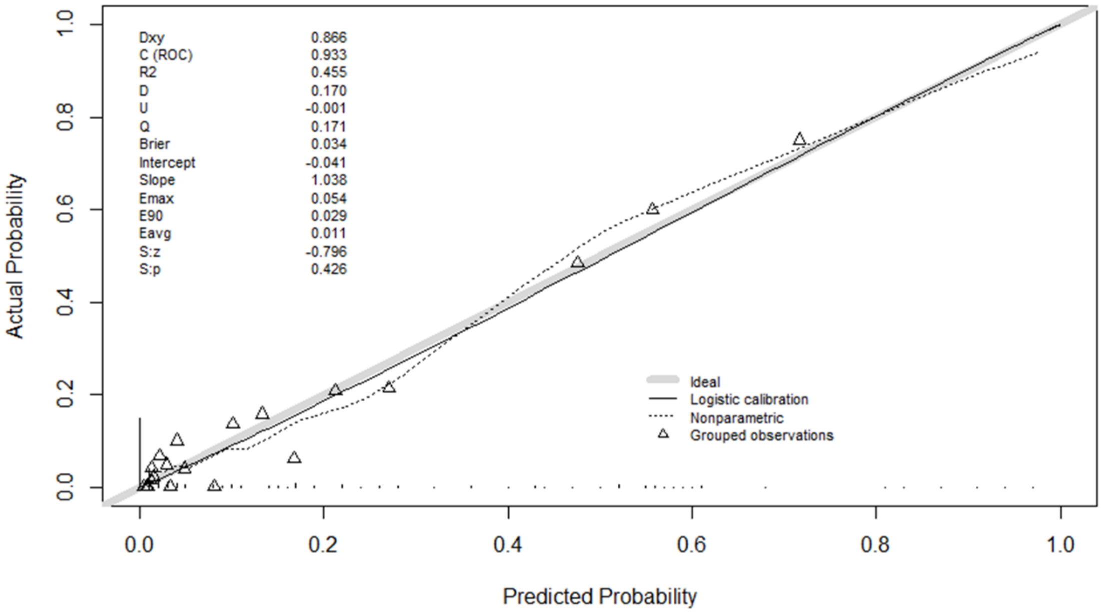

Supplemental Fig. 4 Violin plots of the blood biomarker score in survivors and non-survivors groups.

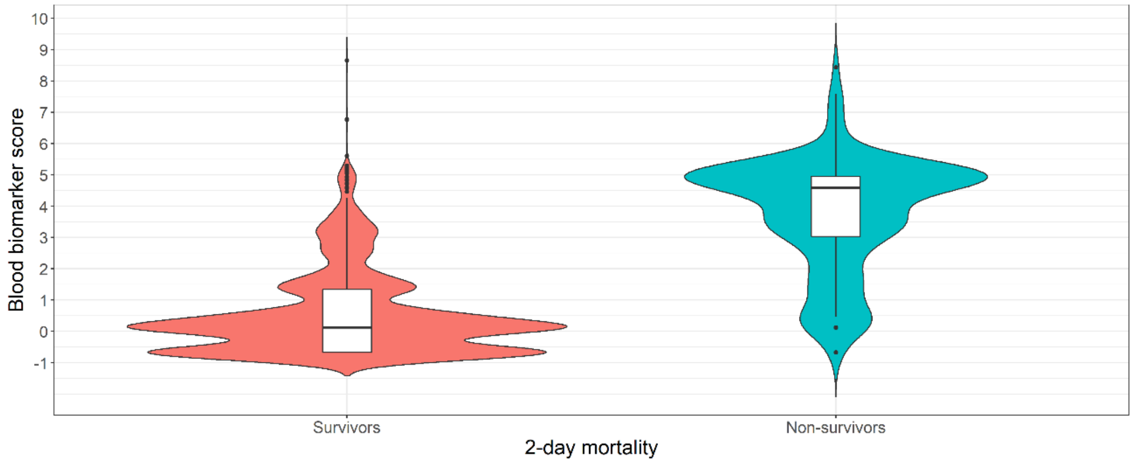

4. Performance comparison

4.1. Statistical accuracy *versus* clinical applicability of the biomarker score

On one hand, predictive models, such as the blood biomarker score for 2-day mortality proposed in this study, must be as accurate as possible. On the other hand, it is important to maximize the clinical applicability of a risk score. In this respect, Zhang et al (2017) proposed a step-by-step tutorial for developing scoring systems for risk stratification based on risk points of input variables. This methodology requires: (*i*) to first convert continuous variables into categorical variables through LOESS curves; (*ii*) to round the coefficients of the LR model to the next integer in order to obtain risk points for each level of the categorical variables. In this way, it is possible to interpret the influence of the values of each variable in the risk score, as well as in the probability of the outcome. However, the discretization of input variables, such as the blood biomarkers, as well as the rounding of the coefficients of the logistic regression model can make a score less accurate. For these reasons, we have assessed the following biomarker score designs:

- C+R blood biomarker score: score built through the categorization (C) of input blood biomarkers (pCO_2, lactate, and creatinine) and rounding (R) of the coefficients of the logistic regression model to the next integer. This configuration maximizes the clinical interpretability and applicability of the score.
- nC+nR score: score obtained as the output of the LR model designed from the continuous blood biomarkers. That is to say, no categorization and rounding

operations are applied. This configuration maximizes the statistical accuracy of the score.

- C+nR score: score built through the categorization (C) of input blood biomarkers and without rounding (nR) the coefficients of the LR model. This configuration represents a tradeoff between statistical accuracy and clinical applicability.

Supplementary Table 2 compares the results obtained with these configurations. It can be seen that there is not a high variation in the results among the three configurations. However, the C+nR blood biomarker score achieved the highest performance in terms of AUC, Brier score, and R^2. For this reason, we chose the C+nR configuration for the blood biomarker score. The proposed score can be interpreted in terms of the blood biomarkers and is easy to calculate in critical situations, as it just requires summing three float numbers obtained in a table.

Supplemental Table 2. Comparison of the performance of different blood biomarker design strategies.

	AUC	Brier score	R^2
**C+R blood biomarker score**	0.925	0.036	0.422
**nC+nR blood biomarker score**	0.915	0.037	0.413
**C+nR blood biomarker score**	0.933	0.034	0.455

Abbreviations: C+R blood biomarker score: score built through the categorization (C) of input blood biomarkers (pCO_2, lactate, and creatinine) and rounding (R) of the coefficients of the logistic regression model to the next integer; nC+nR score: score obtained as the output of the LR model designed from the continuous blood biomarkers; C+nR score: score built through the categorization (C) of input blood biomarkers and without rounding (nR) the coefficients of the LR model.

4.2. Inclusion of age and sex in the blood biomarker score

The biomarker score was only designed with variables from prehospital blood analysis (pCO_2, lactate, and creatinine). However, age and sex are also common variables that can be used in early warning scores and are available in the dataset. Accordingly, we have assessed the effect of incorporating age and sex to the biomarkers score. Supplementary Table 3 shows the comparison of the performance of the blood biomarker score (pCO_2, lactate, and creatinine) with a biomarker score that also integrates age and sex (blood-demographics biomarker score) in terms of AUC, Brier score and R^2. It can be seen that the blood biomarker score achieves a similar performance than the blood-demographics biomarker score in terms of AUC, Brier score, and R^2, which suggests that age and sex do not provide additional information to pCO_2, lactate, and creatinine.

4.3. Logistic regression *versus* decision trees

Decision trees (DT) are a widely used artificial intelligence technique for building interpretable predictive models. A DT model is recursively obtained by the division of a dataset into homogeneous groups using a hierarchical, tree-like structure consisting of consists of a root node, branches, internal nodes, and leaf nodes. In our case, a DT model has been fitted to predict 2-day mortality from categorized blood biomarkers ($pCO2$, lactate, and creatinine) and compared with the blood biomarker score based on logistic regression. Supplementary Table 4 shows the comparison of the performance of the Blood biomarker score based on logistic regression (logistic regression score) and the biomarker score based on decision trees (decision tree score) in terms of AUC, Brier score and R^2. The decision tree score outperformed the logistic regression score in

terms of R^2 (0.464 vs. 0.455). Conversely, the logistic regression score outperformed the decision tree score in terms of AUC (0.933 vs. 0.920) and Brier score (0.034 vs. 0.042). Thus, the application of a decision tree, a more complex predictive algorithm, does not lead to an improvement on the performance.

Supplemental Table 3. Comparison of the performance of the blood biomarker score and the blood-demographics biomarker score.

	AUC	Brier score	R^2
**Blood biomarker score (pCO_2+lactate+creatinine)**	0.933	0.034	0.455
**Blood-demographics biomarker score (pCO_2 + Lactate + Creatinine + Age + Sex)**	0.932	0.036	0.440

Abbreviations: pCO_2: partial pressure of carbon dioxide; AUC: area under the curve of the receiver operating characteristic.

Supplemental Table 4. Comparison of the performance of the logistic regression score and the decision tree score.

	AUC	Brier score	R^2
**Logistic regression score (Blood biomarker score based on logistic regression)**	0.933	0.034	0.455
**Decision tree score (Blood biomarker score based on decision trees)**	0.920	0.042	0.464

SUPPLEMENTAL REFERENCES

20 Zhang Z, Zhang H, Khanal MK. Development of scoring system for risk stratification in clinical medicine: a step-by-step tutorial. Ann Transl Med. 2017; 5(21): p. 436.
